# Pharmacology-informed prediction of the risk posed to fish by mixtures of non-steroidal anti-inflammatory drugs (NSAIDs) in the environment

**DOI:** 10.1016/j.envint.2020.106222

**Published:** 2021-01

**Authors:** Philip Marmon, Stewart F. Owen, Luigi Margiotta-Casaluci

**Affiliations:** a Department of Life Sciences, College of Health, Medicine, and Life Sciences, Brunel University London, London, UB8 3PH, UK; b AstraZeneca, Global Environment, Alderley Park, Macclesfield, Cheshire SK10 4TF, UK

**Keywords:** NSAID, Diclofenac, Predictive toxicology, Fish, Environmental risk assessment, Pharmaceuticals in the environment

## Abstract

The presence of non-steroidal anti-inflammatory drugs (NSAIDs) in the aquatic environment has raised concern that chronic exposure to these compounds may cause adverse effects in wild fish populations. This potential scenario has led some stakeholders to advocate a stricter regulation of NSAIDs, especially diclofenac. Considering their global clinical importance for the management of pain and inflammation, any regulation that may affect patient access to NSAIDs will have considerable implications for public health. The current environmental risk assessment of NSAIDs is driven by the results of a limited number of standard toxicity tests and does not take into account mechanistic and pharmacological considerations. Here we present a pharmacology-informed framework that enables the prediction of the risk posed to fish by 25 different NSAIDs and their dynamic mixtures. Using network pharmacology approaches, we demonstrated that these 25 NSAIDs display a significant mechanistic promiscuity that could enhance the risk of target-mediated mixture effects near environmentally relevant concentrations. Integrating NSAIDs pharmacokinetic and pharmacodynamic features, we provide highly specific predictions of the adverse phenotypes associated with exposure to NSAIDs, and we developed a visual multi-scale model to guide the interpretation of the toxicological relevance of any given set of NSAIDs exposure data. Our analysis demonstrated a non-negligible risk posed to fish by NSAID mixtures in situations of high drug use and low dilution of waste-water treatment plant effluents. We anticipate that this predictive framework will support the future regulatory environmental risk assessment of NSAIDs and increase the effectiveness of ecopharmacovigilance strategies. Moreover, it can facilitate the prediction of the toxicological risk posed by mixtures via the implementation of mechanistic considerations and could be readily extended to other classes of chemicals.

## Highlights

• The current environmental risk assessment does not include mechanistic evaluations.
• We developed a pharmacology-informed framework to predict the risk of 25 NSAIDs.
• The mechanistic promiscuity of the 25 NSAIDs enhances the risk of mixture effects.
• Exposure to NSAIDs in the environment may cause adverse effects in wild fish.
• Risk is increased by high population density, drug use, and low dilution of waste-water effluents.

## Graphical abstract

**Figure:**
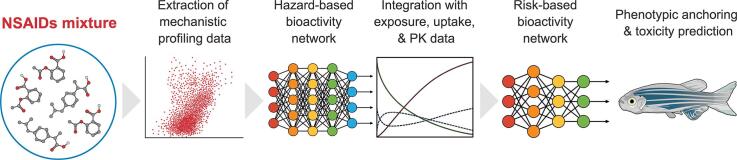

## 1 Introduction

Millions of people worldwide use non-steroidal anti-inflammatory drugs (NSAIDs) to treat a wide variety of health conditions involving inflammation and pain (Gunaydin and Bilge, 2018). One of the consequences of such widespread therapeutic use is that subsequent to excretion from the human body, NSAIDs and their metabolites enter the domestic waste-waters and can reach the aquatic environment where they are detected at low concentrations (Aus der Beek et al., 2016, Lonappan et al., 2016). Administration of NSAIDs to humans, especially when long-term, is associated with an increased risk of adverse events in multiple organs/systems, including gastrointestinal and cardiovascular systems (Conaghan, 2012, Fanelli et al., 2017). These safety concerns led to various regulatory actions during the last twenty years in both North America and Europe, which required drug manufacturers to update product labels with explicit warnings that NSAIDs may increase the risk of serious adverse events (e.g. UK Medicines and Healthcare products Regulatory Agency, 2007, UK Medicines and Healthcare products Regulatory Agency, 2012, US Food and Drug Administration, 2015). In parallel with the clinical safety considerations, the presence of low, but sustained, concentrations of NSAIDs in the aquatic environment has raised the concern that chronic exposure to these compounds may also cause adverse effects in wild fish populations. In 2015, this concern triggered regulatory action and one specific NSAID, diclofenac, was included in the European Union (EU) Watch List of emerging pollutants under the European Water Framework Directive (European Commission, 2015). Diclofenac was subsequently removed from the Watch List in 2018 (European Commission, 2018) once a larger volume of high-quality monitoring data was gathered to allow a refined risk assessment. However, the regulatory and academic discussions concerning the environmental risk assessment (ERA) of NSAIDs continued and have reached the point that some stakeholders are advocating a stricter regulation of over-the-counter NSAIDs, such as diclofenac, and even the substitution with compounds associated with a lower environmental risk (OECD, 2019).

Considering the global clinical importance of NSAIDs for the management of pain and inflammation, any regulation that may affect patient access to NSAIDs will have considerable implications for public health. Thus, it is of paramount importance that all relevant scientific evidence, beyond the boundaries of ecotoxicology, is used to inform regulatory decision-making. The inclusion of diclofenac in the EU Watch List highlighted three potential limitations of the current risk assessment of NSAIDs. Firstly, from a toxicological perspective, the original decision to include diclofenac in the list was driven by a relatively small set of experimental data (e.g. Hoeger et al., 2005, Mehinto et al., 2010; Schwaiger et al., 2004; Triebskorn et al., 2004) concerning chronic effects in fish species, which were subsequently the object of scientific debate (Memmert et al., 2013). The reasons underlying the debate were not related to the widely accepted notion that diclofenac may trigger adverse effects in fish (hazard assessment), but rather to the degree of reproducibility of the experiments that characterised those effects, and to the range of environmental concentrations that may trigger them (risk assessment). Secondly, the current ERA of diclofenac (and any other pharmaceutical) does not incorporate mechanistic and mode-of-action considerations, limiting the potential to implement predictive toxicology approaches to support decision-making. Finally, more than 20 different NSAIDs are currently available on the market, and all of them exert their pharmacological effects by inhibiting one or both isoforms of the enzyme cyclooxygenase (COX-1 and COX-2). This pharmacological aspect implies that diclofenac may not be the only NSAID of concern, and that mixture effects might occur.

To overcome these challenges, we developed a novel pharmacology-informed framework that enables the prediction of the risk posed to fish by NSAIDs and their mixtures under realistic exposure scenarios. Our framework is centred on the integration of two mechanistic perspectives, network-centred and target-centred, and on the consideration of drug concentrations inside the organism (rather than in the surrounding water) as an essential parameter for the generation of accurate and realistic risk predictions. This research aims at providing a valuable tool that can facilitate the implementation of mechanistic considerations into the future regulatory environmental risk assessment of NSAIDs and ecopharmacovigilance strategies.

## 2 Methods

### 2.1 Compound identification

To identify the NSAIDs currently present on the market, we screened the database DrugBank (www.drugbank.ca; Wishart et al., 2018) and selected all pharmaceuticals labelled as ”COX-inhibitor” or “NSAID”. The physico-chemical properties of each compound – including LogK_OW_ and LogD_7.4_ - were retrieved from the database ChemSpider (www.chemspider.com).

### 2.2 Prediction of blood concentrations of NSAIDs in wild fish

Measured surface water concentrations for each compound were retrieved from a database curated by the German Environment Agency (Umweltbundesamt – UBA) (https://www.umweltbundesamt.de/en/database-pharmaceuticals-in-the-environment-0). At the date of access (November 2019), the database contained environmental concentrations of human and veterinary pharmaceutical residues in 53 environmental matrices from 75 countries, extracted from 1519 publications, and 240 review articles (Eike et al., 2019). Measured concentrations in UK freshwaters were used as an example of environmentally realistic exposure scenario. Specifically, we used the highest average measured concentrations in treated waste-water treatment plant (WWTP) effluents and surface waters (i.e. freshwaters). These water concentrations were subsequently used to predict the concentration of each compound in the blood of wild fish by applying the Fish Plasma Model, as described by Margiotta-Casaluci et al., 2014, Margiotta-Casaluci et al., 2016 (Supplementary Table 1).

### 2.3 Network-centred approach

The network-centred approach was driven by the hypothesis that NSAID-mediated adverse effects are induced through the perturbation of a network of drug targets (i.e. drug polypharmacology and bioactivity profile).

### 2.4 Extraction of drug-target interaction and *in vitro* bioactivity profiling data

*In vitro* bioactivity profiling data for 25 different NSAIDs was extracted from two sources: 1) the ‘US Environmental Protection Agency (US EPA) Toxicity Forecaster (ToxCast) database (U.S. EPA. 2015. ToxCast & Tox21 Summary Files from invitrodb_v3.2. Retrieved from https://www.epa.gov/chemical-research/toxicity-forecaster-toxcasttm-data between May 2019 and October 2019. Data released May 2018) (Williams et al., 2017), and 2) the European Bioinformatics institute (EBI) ChEMBL database (http://www.ebi.ac.uk/chembl; Gaulton et al., 2017). The data extracted from ToxCast included drug target identifier and drug concentration at 50% maximum activity (AC50). Data extraction was limited to the interactions labelled as ‘*active*’, hence, those labelled as ‘*inactive*’ were excluded from the analysis. On the other hand, the data extracted from ChEMBL included drug target identifier and half-maximum inhibitory concentrations (IC50). Similarly, in this case, data extraction was limited to the interactions labelled as ‘active’, whereas those labelled as ‘*not active*’ or ‘*not determined’* were excluded from the analysis. The bioactivity profiling data used in this study is available in Supplementary File 1.

### 2.5 Data harmonisation and processing

The data extracted from ToxCast and ChEMBL were manually harmonised to ensure inter-database comparability and maximise data usability. AC50 and IC50 values were converted to, and uniformly expressed as, ng/mL. When data from multiple species were available, human data was used as the first choice; if unavailable, rodent data was used instead. When multiple datapoints were available for the same target, the lowest AC50 (or IC50) value was selected for the final analysis. ToxCast and ChEMBL use different target annotation strategies, hence all drug target identifiers were converted into human gene symbols to ensure target specificity and allow dataset merging. The gene symbol nomenclature was harmonised using GeneCards as the reference source (GeneCards.org; Stelzer et al., 2016). The harmonised datasets from ToxCast and ChEMBL were finally combined to assess the database-specific bioactivity coverage (i.e. degree of overlap between ToxCast and ChEMBL interactions), after which duplicate interactions were removed. As before, the lowest AC50 (or IC50) value for each target was retained for the final analysis. This process led to the generation of combined ToxCast/ChEMBL drug-target interaction profiles (Supplementary File 1), which were used in the subsequent network analyses.

### 2.6 Generation of hazard-based and risk-based drug-target interaction networks

Drug-target interaction networks were generated using the Cytoscape software (Shannon et al., 2003). The initial network included all the drug-target interactions present in our database, irrespective of any effect concentration data. For this reason, this network represented a hazard bioactivity network, which was used as the point of departure for the subsequent analyses. To determine the meaningfulness of the network under realistic exposure scenarios, each drug-target interaction node was filtered using the drug concentrations predicted to be present in the blood of wild fish in the UK (i.e. using the highest average measured concentrations in treated WWTP effluents). Using this approach, the refined network contained only those interactions that occur at concentrations equal to, or lower than, the exposure levels of interest. To evaluate the impact of integrating exposure data within the network, we simulated three different exposure scenarios: highest average measured concentrations in UK effluents, as well as 10-fold and 100-fold above those concentrations.

### 2.7 Target-to-phenotype analysis

To predict the phenotypic meaning of the risk-based drug-target interaction network (i.e. the one occurring at realistic exposure scenario only), we first identified the gene involved in each interaction (e.g. cyclooxygenase 1 inhibition → PTGS1 gene), and subsequently we performed a gene-phenotype anchoring analysis using the Monarch Initiative platform (www.monarchinitiative.org). The latter is a “*collaborative, open science effort that aims to semantically integrate genotype–phenotype data from many species and sources in order to support precision medicine, disease modelling, and mechanistic exploration*” (Mungall et al., 2017). Using Monarch, we extracted all the available zebrafish-specific phenotypic data associated with alterations of the target genes (e.g. mutations, variants, artificial alterations such as knock out or knock down). This analysis generated an array of phenotypes that might potentially occur in wild fish under the considered exposure scenario (i.e. highest average measured concentrations in treated WWTP effluents in the UK).

### 2.8 Target-centred approach

The target-centred approach was driven by the hypothesis that NSAID-mediated adverse effects are induced by the inhibition of COX-1 and COX-2, which are the primary targets of NSAIDs.

### 2.9 Literature review and data extraction

To identify all the relevant effects caused by NSAIDs in fish, we performed a literature review to identify relevant medium-to-long term *in vivo* freshwater ecotoxicity studies (4-days or longer). The literature search was conducted via PubMed and Google Scholar using a combination of keywords (e.g. drug name, endpoint name, species, toxicity) and was restricted to English language publications only. Statistically significant effect data was extracted from each paper. Whenever available, we also extracted the average value for each parameter and the relative uncertainty measure (e.g. standard deviation) to calculate the effect size reported in each study. For the studies that reported multiple concentration and/or time responses, each dose and/or time point was considered as an independent data point in the database. Other extracted information included exposure concentrations, duration of exposure, fish species, life stage, and effect direction (increase or decrease). A quality assessment of all extracted data and relative database was performed by two different operators to evaluate the consistency between extracted data and original values. Considering the highly variable vocabulary used in different papers (e.g. same endpoint defined using different terms), we carried out a harmonisation process to ensure data comparability.

### 2.10 Prediction of internal effect concentrations and equivalence calculation

To account for the different uptake profile of each drug, we transformed water exposure concentrations for all the identified drug-effect combinations into predicted effect plasma concentrations using the Fish Plasma Model as described by Margiotta-Casaluci et al., 2014, Margiotta-Casaluci et al., 2016. Considering the hypothesis that NSAID-induced effects are mediated by COX-1 and COX-2 inhibition, and that all NSAIDs act via inhibiting COX-1 and/or COX-2, we expressed each drug plasma concentration as equivalent to a reference NSAID (i.e. diclofenac). To do so, we considered the COX-1 inhibition IC50 of diclofenac as the reference value (=1); successively, we calculated a “diclofenac-equivalence conversion factor” for every other NSAID using the formula “COX-1-inhibition IC50_(diclofenac)_ / COX-1-inhibiton IC50(_other NSAID_)”. The resulting conversion factor was used to express all NSAIDs plasma concentrations as “diclofenac-equivalent plasma concentrations”. The focus on COX-1 rather than COX-2 was justified by two observations: a) all NSAIDs tested *in vivo* were dual COX inhibitors; b) in human pharmacology, COX-1 inhibition is considered to be the main driver of NSAIDs-mediated side effects, as COX-2 is generally expressed at low levels, and is only induced when the organism is experiencing an ongoing inflammation (Rouzer and Marnett, 2009).

### 2.11 Generation of a multi-scale COX-1-centred model to predict the risk of *in vivo* chronic effects

The data described above was integrated to generate a multi-scale model displaying the range of NSAID plasma concentrations (expressed as diclofenac-equivalents calculated using “diclofenac human COX-1 IC_50_” as the reference value), associated with mode-of-action-relevant adverse effects, under medium/long-term exposure scenarios. To facilitate the interpretation of the model and its relevance for the ERA process, we incorporated three threshold levels. Two of these thresholds represent the concentration of the NSAIDs mixture predicted to occur in the plasma of wild fish in a) UK WWTP effluents, and b) UK surface waters. The third threshold level represents the range of predicted NSAIDs plasma concentrations that are likely to induce mortality.

## 3 Results

### 3.1 NSAIDs selection and environmental occurrence

25 NSAIDs were identified in the DrugBank database: amfenac, aspirin, carprofen, celecoxib, diclofenac, etodolac, etoricoxib, flufenamic acid, flurbiprofen, ibuprofen, indomethacin, ketoprofen, ketorolac, mefenamic acid, meloxicam, naproxen, niflumic acid, nimesulide, oxaprozin, piroxicam, rofecoxib, sulindac, tenoxicam, tolfenamic acid, valdecoxib. Four of these compounds were classified as COX-2-selective inhibitors (celecoxib, etoricoxib, rofecoxib, and valdecoxib), whereas 21 compounds were classified as non-selective COX inhibitors. According to the UBA database of pharmaceuticals in the environment, 19 out of 25 NSAIDs were detected in the aquatic environment, in 66 different countries around the world, supporting our hypothesis that the overall environmental risk of NSAIDs should be addressed from a mixture perspective. This data mining exercise revealed a wide range of concentrations detected worldwide in surface waters and waste-water treatment plant (WWTP) effluents (Supplementary Figure 1); however, when multiple measurements for the same compounds were available, the observed median value was generally below 1 µg/L. Carrying out a detailed analysis of the environmental levels of NSAIDs is beyond the scope of the present work. Hence, for the next steps of our analysis we only considered the concentrations of NSAIDs measured in UK surface waters and WWTP effluents as the default exposure scenario for our toxicity predictions (Supplementary Table 1). Specifically, 7 out of 25 NSAIDs were detected in UK WWTP effluents, and 6 in surface waters. These numbers are in line with the number of NSAIDs detected in other countries characterised by intensive environmental monitoring activity (e.g. Canada, USA, Germany, Sweden, Japan). It is important to note that the exposure concentrations were selected to represent a worst-case scenario in the UK. For example, based on the data generated from two large UK-wide waste-water treatment plant monitoring programmes, Comber et al. (2018) estimated a diclofenac median effluent concentration equal to 0.33 µg/L, whereas the 95th percentile is 0.5 µg/L. As a term of comparison, the effluent concentration of diclofenac used in our simulation was 0.42 µg/L, indicating a good degree of agreement with other worst-case scenarios estimated in other studies.

### 3.2 Analysis of the primary pharmacological activity of NSAIDs

**Fig. 1:**
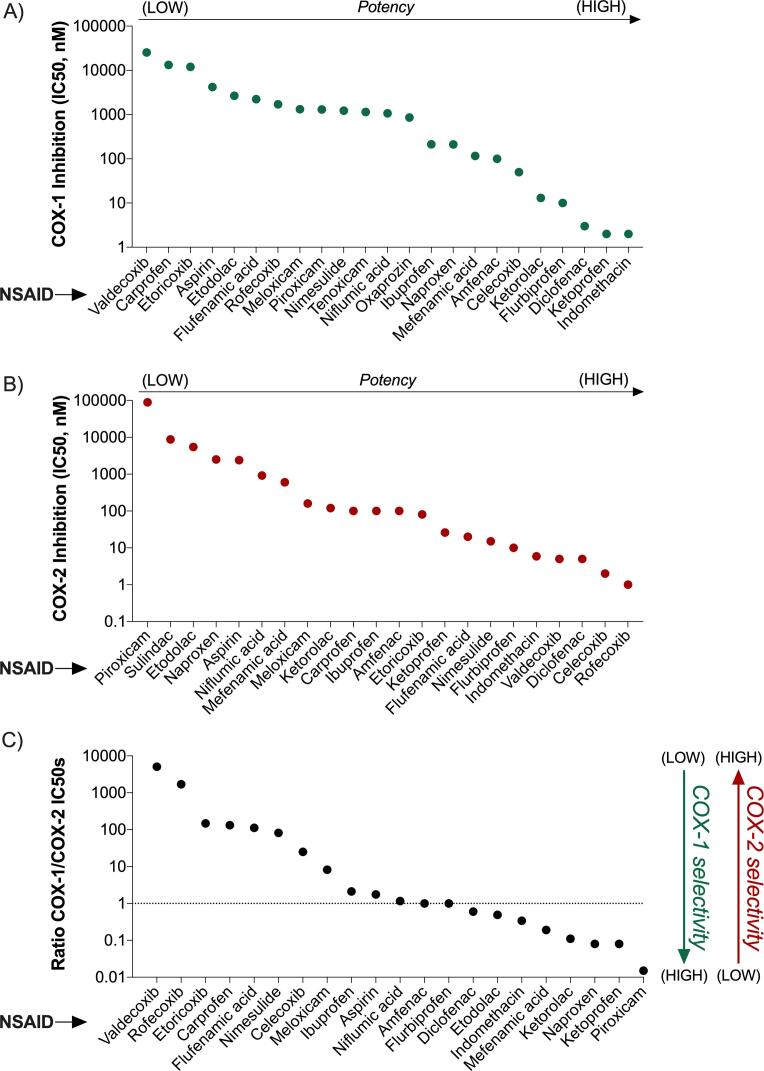
**Pharmacological activity of NSAIDs on the primary targets COX-1 and COX-2**. A) Lowest COX-1 IC50 values retrieved from ToxCast/ChEMBL. B) Lowest COX-2 IC50 values retrieved from ToxCast/ChEMBL. C) Ratio of COX-1/COX-2 IC50s, indicating the selectivity of each compound towards either COX-1 or COX2.

The inhibition of COXs is the primary mechanism of action of NSAIDs. The analysis of COXs-inhibitory activity of the 25 compounds revealed a wide range of pharmacological potencies (Fig. 1). IC50 values for COX-1 inhibition ranged from 2 to 3 nM (i.e. indomethacin, ketoprofen, diclofenac) to over 25,000 nM (i.e. valdecoxib) (Fig. 1A). Similarly, IC50 values for COX-2 inhibition ranged from 1 to 2 nM (i.e. rofecoxib, celecoxib) to over 89,000 nM (i.e. piroxicam) (Fig. 1B). The analysis of the ratio between COX-1 and COX-2 inhibition IC50s revealed the selectivity of each compound for the two isoforms of the enzyme (Fig. 1C). Unsurprisingly, COX-2 selective inhibitors such as rofecoxib, valdecoxib, and etoricoxib displayed the highest selectivity for COX-2. These compounds have been specifically developed to display such a pharmacological feature. However, non-selective NSAIDs - such as carprofen, flufenamic acid, nimesulide, and meloxicam – also showed considerable COX-2 selectivity. Piroxicam was the NSAID with the highest COX-1 selectivity, followed by naproxen and ketoprofen (Fig. 1C).

The AC50 and IC50 values used in this study were retrieved with the explicit intention to simulate a worst-case scenario (i.e. when multiple values were available, the lowest value was selected for the final analysis). However, it is important to consider that the inter-experiment variability in IC50 values can be considerable. To assess such variability, we compared the ToxCast/ChEMBL data used in our model with two additional IC50 values retrieved from the literature (Supplementary Figure 2). The comparative analysis of COX-1 IC50s confirmed that our data were at the bottom of the variability range, except for flurbiprofen. The analysis of COX-2 IC50 values revealed a less consistent scenario, where ToxCast/ChEMBL values were at the bottom of the variability range in only 7 out of 25 cases. In some cases, the gap between the ToxCast/ChEMBL values and the literature values was considerable (e.g. rofecoxib COX-2 IC50s: 1, 340, 510 nM). Notably, we also observed a surprising variability between the two alternative IC50 values retrieved from Reinsford (2004). It is important to note that those values were generated using different test systems. A similar degree of variability was also observed in the COX-1/COX-2 IC50 ratios, influencing the interpretation of the selectivity of the compound for either COX-1 or COX-2.

### 3.3 Hazard-based bioactivity networks of NSAIDs mixtures

**Fig. 2:**
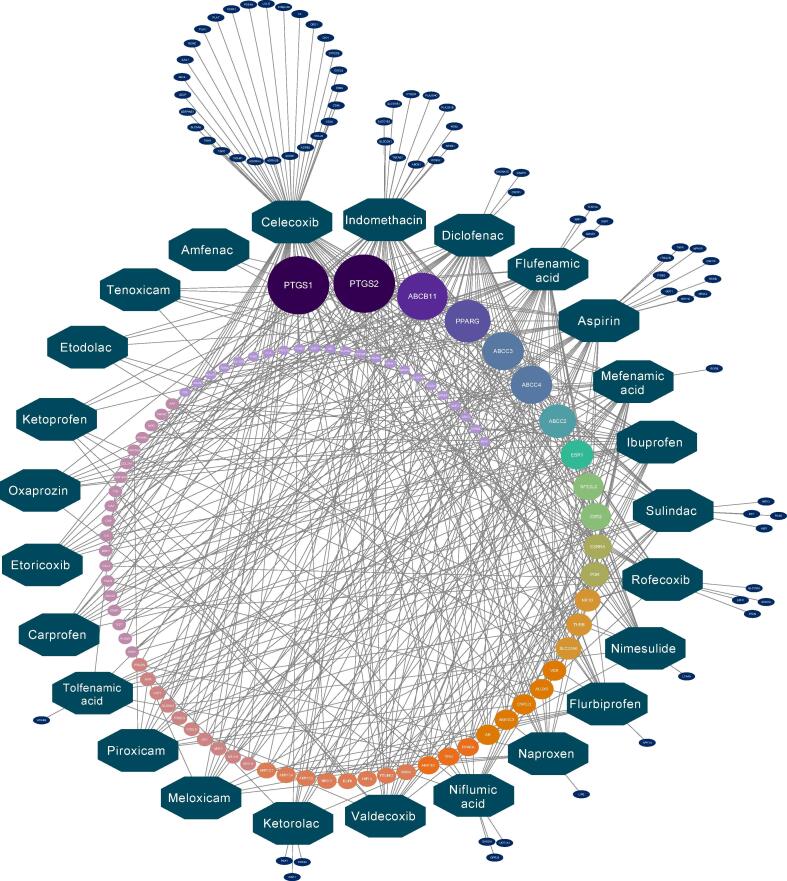
**Drug-target interaction network for a mixture of 25 NSAIDs.** The green octagons indicate the single drugs. The nodes on the external layer indicate the drug-target interactions that are unique for each compound. The nodes in the inner area indicate the drug-target interactions shared by at least two different NSAIDs. The larger the size of the inner nodes, the higher the number of NSAIDs that interact with that target. The different colours of the inner nodes indicate a different number of NSAIDs sharing the target. Each drug-target relationship is associated to a specific AC50 (or IC50) value, retrieved from a combined ToxCast/ChEMBL database. No exposure data is incorporated into this model (e.g. some AC50 or IC50 values represent unrealistic exposure levels); hence, this network can be considered as a hazard-based bioactivity network. Note that most of the targets are shared by two or more NSAIDs, providing a mechanistic rationale for potential NSAIDs-mixture effects beyond COX inhibition (in this network PTGS1 = COX-1, and PTGS2 = COX-2). (For interpretation of the references to color in this figure legend, the reader is referred to the web version of this article.)

Understanding the secondary mechanisms of action of drugs can significantly enhance the prediction of their toxicity profile. To explore the mechanisms of action of NSAIDs beyond COX inhibition, we leveraged the ToxCast and ChEMBL platforms to generate a bioactivity network for the mixture of 25 NSAIDs (Fig. 2). The combination of the ToxCast and ChEMBL databases was aimed at expanding the biological space covered in our analysis. To evaluate this aspect, we analysed the gain in biological space due to the merging exercise (Supplementary Figure 3). The analysis revealed that the degree of overlap between ToxCast and ChEMBL data was minimal (i.e. zero shared interactions for 21 NSAIDs out of 25). The combination of the two data sources allowed us to expand the biological space, while increasing the relevance of the pharmacological network.

The mechanistic analysis revealed that the 25 NSAIDs have a wide range of mechanisms of action beyond COXs inhibition. The number of recorded interactions ranged from 3 to 74 (Fig. 2). The compound with the highest number of interactions was celecoxib (n = 74), followed by indomethacin (n = 47) and diclofenac (n = 40). On the other hand, the compounds with the lowest number of recorded interactions were etodolac (n = 6), tenoxicam (n = 6), and amfenac (n = 3). In total, the mixture of 25 NSAIDs was associated with 507 interactions, involving 157 distinct targets; 83 of these targets were shared by at least 2 NSAIDs, whereas 74 targets were modulated only by individual drugs. PTGS1 and PTGS2 (corresponding to COX-1 and COX-2) were the targets with the highest levels of promiscuity, and were shared by 23 and 22 NSAIDs, respectively. Notably, both ToxCast and ChEMBL did not contain any information concerning the COXs inhibitory activity for two NSAIDs, sulindac and tolfenamic acid, despite the known COX-inhibitory activity of these compounds. After PTGS1 and PTGS2, the targets with the highest levels of promiscuity were the bile salt export pump (ABCB11; shared by 19 NSAIDs) and the peroxisome proliferator-activated receptor gamma (PPARγ; shared by 17 NSAIDs). Other targets shared by 10 or more NSAIDs were the transporters ABCC4 (n = 15), ABCC3 (n = 15) and ABCC2 (n = 13), the estrogen receptors ESR1 (n = 11) and ESR2 (n = 10), and the nuclear factor erythroid 2-related factor 2 (NFE2L2, n = 10). A detailed list of interactions for each target and for each drug is available in Supplementary File 1 whilst the full drug-target interaction network is represented in Fig. 2. Of the 25 NSAIDs, 14 interacted with unique targets that were not shared by any other compound. The drug with the highest number of unique interactions was celecoxib (n = 28), followed by indomethacin (n = 11), and aspirin (n = 9) (Fig. 2). It is important to note that the bioactivity network described above does not include any information about the concentration of the drug needed to modulate each target, hence it should be considered as a hazard network.

### 3.4 Risk-based bioactivity networks of NSAIDs mixtures

**Fig. 3:**
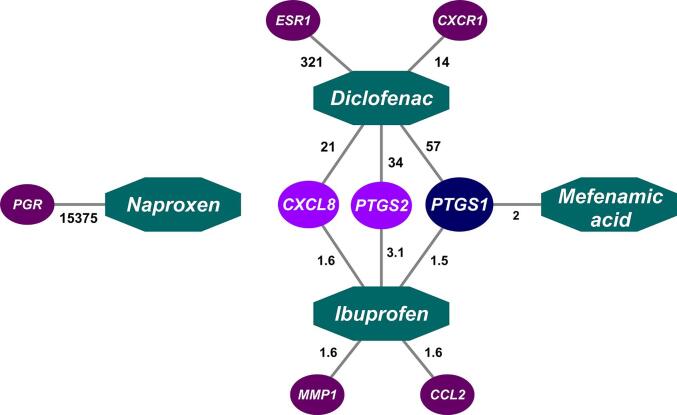
**Drug-target interaction network, for a mixture of 25 NSAIDs, predicted to occur at a worst-case exposure scenario (UK highest average measured concentration in wastewater treatment plants effluents).** The green octagons indicate the single drugs. Drug targets are represented by color-coded nodes. Each colour indicates the different number of drugs that act on the associated target (PTGS1: 3 drugs, PTGS2: 2 drugs, CXCL8: 2 drugs, CCL2: 1 drug, CXCR1: 1 drug, ESR1: 1 drug, MMP1: 1 drug, PGR: 1 drug). The numbers indicated next to each drug-target connection represent the ratio between the ToxCast/ChEMBL AC50 (or IC50) value and the drug concentration predicted to be present in the blood of wild fish in the UK, under the considered exposure scenario. For example, the concentration of diclofenac present in the blood of wild fish is predicted to be 256-times above the considered IC50 for PTGS1. Abbreviations: CCL2: C-C Motif Chemokine Ligand 2; CXCL8: C-X-C Motif Chemokine Ligand 8; CXCR1: C-X-C Motif Chemokine Receptor 1; ESR1: Estrogen Receptor 1; MMP1: Matrix Metallopeptidase 1; PGR: Progesterone Receptor; PTGS1: Prostaglandin-Endoperoxide Synthase 1; PTGS2: Prostaglandin-Endoperoxide Synthase 2. (For interpretation of the references to color in this figure legend, the reader is referred to the web version of this article.)

To interpret the environmental relevance of the hazard bioactivity network, we filtered each drug-target interaction using the concentrations of NSAIDs predicted to be present in the blood of wild fish in the UK. The resulting network displays only the drug-target interactions predicted to occur at the defined exposure scenario (i.e. highest average measured concentrations in UK WWTP treated effluents) (Fig. 3) and can be considered as a risk-based network. In the specific example used here, the refined network suggests that only 8 targets are likely to be modulated in wild fish exposed to those effluent concentrations: C-C motif chemokine 2 (CCL2), interleukin-8 (CXCL8), C-X-C chemokine receptor type 1 (CXCR1), estrogen receptor 1 (ESR1), progesterone receptor (PGR), interstitial collagenase (MMP1), prostaglandin G/H synthase 1 (PTGS1), and prostaglandin G/H synthase 2 (PTGS2) (note that the latter two targets correspond to COX-1 and COX-2). Three out of 8 targets are shared by multiple NSAIDs, whereas the other five targets are only modulated by single drugs. To identify the drivers of the risk within the interaction network, we calculated the ratio between predicted blood concentrations and the AC50 (or IC50) values associated with each drug-target interaction. The analysis showed that the targets with the highest risk are the two steroid receptors PGR and ESR1, as blood concentrations of naproxen and diclofenac were predicted to be 15,375-fold and 321-fold higher than the drug-specific AC50 values. These high values were driven by the low ToxCast AC50s reported for naproxen-induced PGR modulation and diclofenac-induced ESR1 modulation, which were 0.007 nM and 0.5 nM, respectively. The data for PGR was generated employing a GAL4 β-lactamase reporter gene technology using PR-UAS-bla HEK 293 T cells, whereas the data for ESR1 was generated with a luciferase-coupled ATP quantitation technology using human breast tissue cells. Diclofenac was also the driver of the risk for modulation of PTGS1 (ratio = 57), PTGS2 (ratio = 34), CXCL8 (ratio = 21), and CXCR1 (ratio = 14).

### 3.5 Phenotypic anchoring of the risk-based bioactivity network

To elucidate the phenotypic relevance of the targets displayed in the risk-based network (Fig. 3), we performed a gene-phenotype association analysis by data-mining available databases. The analysis generated a list of highly specific zebrafish phenotypes that may be observed following perturbation of the 8 targets of interest (Table 1). These phenotypes indicate that the risk-based NSAIDs bioactivity network may lead to profound effects on general development; the cardiovascular and immune systems; the liver, pancreas, and kidneys; growth and reproduction. It is important to note that the effects on development, growth, and reproduction have high regulatory relevance as they are considered as apical endpoints that, in turn, may perturbate population dynamics. From a risk-assessment perspective, this analysis cannot provide quantitative indications on the likelihood that each phenotype may occur. However, it provides a highly granular prediction of the endpoints that could be used for a potential experimental assessment of the case-specific risk.

**Table 1:** Zebrafish-specific phenotypes associated with the perturbation of the NSAIDs-targets predicted to be modulated at environmentally relevant exposure scenarios (i.e. UK). (Targets: CXCL8, CXCR1, ESR1, MMP1, PGR, PTGS1, PTGS2 (no data available for CCL2)).

**Target**	**Function/system/organ**	**Phenotype**	**Phenotype ID**
PTGS1	Development	Abnormal otolith in otic vesicle	ZP_0003813
ESR1	Development	Altered sex ratio	ZP_0103077
MMP1	Development	Curled notochord	ZP_0005644
PTGS1, PTGS2	Development	Disrupted cilium development	ZP_0018462
PTGS1	Development	Disrupted gastrulation	ZP_0000567
ESR1	Development	Disrupted neuromast development	ZP_0001566
PTGS1	Development	Disrupted skeletal muscle plasticity	ZP_0100172
PTGS1, PTGS2	Development	Hydrocephalus	ZP_0018285
MMP1	Development	Hyperplastic epithelium	ZP_0005645
CXCL8	Development	Increased progenitor cells	ZP_0022176
MMP1	Development	Kinked post-vent region	ZP_0001145
MMP1	Development	Malformed caudal fin actinotrichia	ZP_0005646
MMP1, PTGS1	Development	Ventrally curved trunk	ZP_0000636
MMP1	Development	Yolk sac oedema	ZP_0002060
PTGS1	Cardiovascular system	Abnormal heart symmetry	ZP_0002925
MMP1	Cardiovascular system	Decreased blood flow	ZP_0003573
PTGS1, PTGS2	Cardiovascular system	Decreased hematopoietic stem cells	ZP_0000022
PTGS1	Cardiovascular system	Disrupted heart looping	ZP_0002506
CXCL8	Cardiovascular system	Disrupted vasculogenesis	GO_0001570
CXCL8	Cardiovascular system	Increased hematopoietic stem cells	ZP_0021393
MMP1	Cardiovascular system	Pericardial oedema	ZP_0000038
PTGS1	Reproduction	Decreased egg viability	ZP_0000212
ESR1	Reproduction	Decreased testis size	ZP_0019448
PGR	Reproduction	Disrupted ovulation	ZP_0017606
PGR	Reproduction	Disrupted reproduction	ZP_0017607
PGR	Reproduction	Increased ovary size	ZP_0019913
PGR	Reproduction	Sterile female	ZP_0004113
CXCL8, CXCLR1	Immune system	Abnormal leukocyte migration	GO_0002523
MMP1	Immune system	Abnormal macrophage chemotaxis	GO_0048246
CXCL8, CXCLR1	Immune system	Abnormal response to bacteria	GO_0009617
CXCL8, CXCLR1	Immune system	Abnormal response to wounding	GO_0009611
CXCR1	Immune system	Decreased neutrophil number	ZP_0011617
MMP1	Growth	Decreased trunk size	ZP_0000027
PGR	Growth	Increased trunk size	ZP_0014050
PGR	Growth	Increased weight	ZP_0015745
PTGS1	Liver	Abnormal liver	ZP_0018785
PTGS1, PTGS2	Liver	Decreased liver size	ZP_0000720
PTGS1, PTGS2	Pancreas	Decreased exocrine pancreas size	ZP_0002701
PTGS1	Kidney	Abnormal pronephric distal late tubule	ZP_0019006

### 3.6 Multi-scale COX-1-centred model to predict the risk of *in vivo* chronic effects

**Fig. 4:**
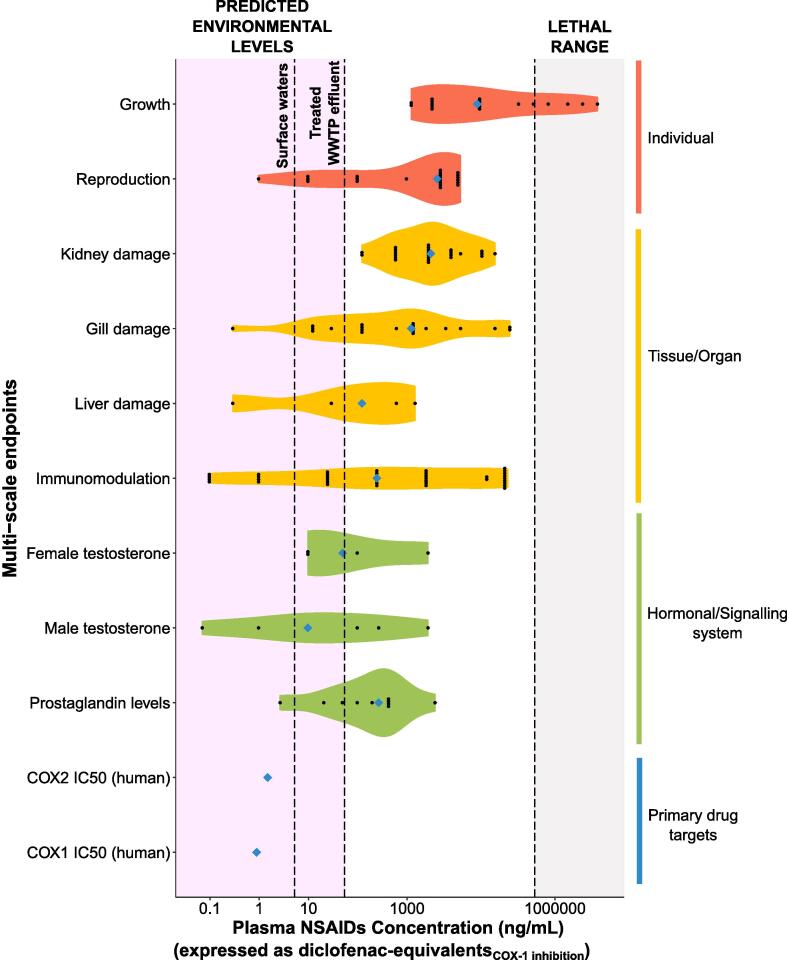
**Predicted NSAID plasma concentrations associated with the manifestation of adverse phenotypes at multiple levels of biological organisation.** NSAID plasma concentrations are expressed as ‘Diclofenac-equivalents_(COX-1inhibition)_ (ng/mL)’. Each violin displays the distribution of plasma concentrations that caused a statistically significant effect in *in vivo* studies that involved the exposure of fish species to NSAIDs, for a minimum of 4 days and a maximum of 132 days. The 151 experimental data points portrayed in the graph were retrieved from 26 studies published between 2004 and 2018. The dotted line on the right represents the plasma concentration of ‘diclofenac-equivalents’ associated with mortality. The dotted vertical lines on the left indicate the environmental levels of the mixture of NSAIDs detected in the UK (i.e. the highest measured average concentrations in surface waters, and waste-water treatment plant effluents). These lines can be used to interpret the environmental relevance of the effect data and the related risk. This exposure scenario should only be used as example to demonstrate the application of the model for risk assessment. A more detailed analysis of the environmental levels of NSAIDs should be carried out to conduct a formal risk assessment.

The gene-phenotype association analysis, described above, provides a solely qualitative result. To overcome this challenge and provide a quantitative estimation of the toxicological risk, we generated a multi-scale model portraying the range of blood concentrations of NSAIDs (expressed as diclofenac-equivalents, ng/mL - calculated using “diclofenac human COX-1 IC_50_” as the reference value) associated with statistically significant adverse phenotypes; under conditions of medium-to-long term exposure (longer than 4 days) (Fig. 4). The model was based on 151 data points generated in 26 *in vivo* studies, carried out using 10 different fish species (Bhandari and Venables, 2011, Bickley et al., 2017, Collard et al., 2013, Flippin et al., 2007, Ghelfi et al., 2016, Gröner et al., 2017, Han et al., 2010, Hoeger et al., 2005, Ji et al., 2013; Lister and Van Der Kraak, 2008; Mathias et al., 2018, Mehinto et al., 2010, Memmert et al., 2013, Morthorst et al., 2013, Morthorst et al., 2018; Näslund et al., 2017; Pandey et al., 2017, Patel et al., 2016, Praskova et al., 2014, Ribas et al., 2017, Saravanan et al., 2012, Schwaiger et al., 2004, Stancova et al., 2015, Triebskorn et al., 2004, Yokota et al., 2016, Yokota et al., 2017). The data included 9 different types of *in vivo* effect, at various level of biological organisation, such as: prostaglandin levels, male and female testosterone, immunomodulation, liver damage, gill damage, kidney damage, reproduction, and growth. To facilitate the interpretation of the data, we incorporated three different reference concentrations (threshold levels) into the model: 1) The lethal range of blood concentrations starting at 388,105 ng/mL diclofenac-equivalents; 2) the predicted plasma levels of the mixture of 7 NSAIDs, detected in UK WWTP effluents, corresponding to 54 ng/mL diclofenac-equivalents; 3) the plasma levels of the mixture of 7 NSAIDs, detected in UK surface waters (i.e. freshwaters), corresponding to 5.2 ng/mL diclofenac-equivalents. A total of 46 out of 152 effect data points corresponded to plasma concentrations lower than 54 ng/mL diclofenac-equivalents (exposure scenario considering UK WWTP effluents); whereas only 14 out of 152 data points corresponded to plasma concentrations lower than 5.2 ng/mL diclofenac-equivalents (exposure scenario considering UK surface waters). The conversion factors used to convert all relevant NSAIDs into diclofenac-equivalents are provided in Supplementary File 1.

**Fig. 5:**
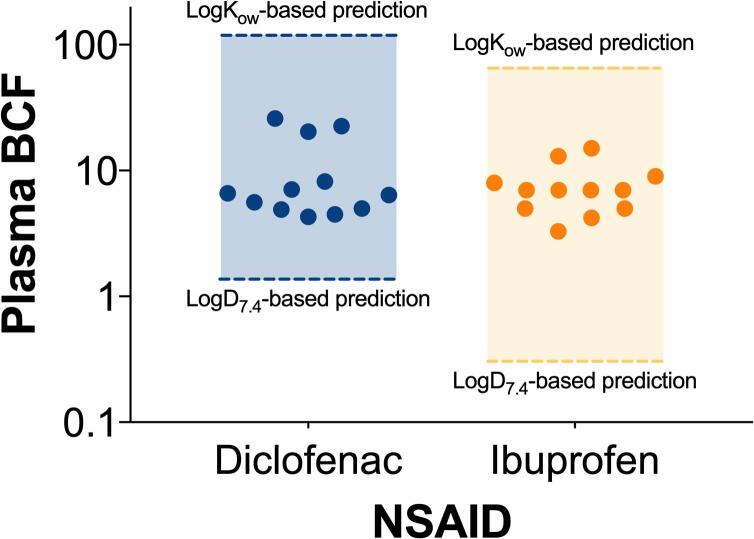
**Predicted versus measured plasma BCF.** The range of predicted values were generated by considering both Log K_OW_ and Log D_7.4_ as the input parameters of the fish plasma model. Measured BCF values were retrieved from four *in vivo* studies published in the literature (Bickley et al., 2017; Brown et al., 2007; Cuklev et al., 2011, Lahti et al., 2011).

The analyses provided here were based on the assumption that the Fish Plasma Model represents a reliable tool to predict the plasma concentration of drugs in adult fish. To validate this assumption, we screened the literature to identify a set of experimentally determined plasma bioaccumulation factors (plasma BCF) for diclofenac and ibuprofen (Bickley et al., 2017, Brown et al., 2007; Cucklev et al., 2011; Lahti et al., 2011). The comparison of this data with the plasma BCFs predicted by the Fish Plasma Model revealed that the experimental values were always within the range of concentrations predicted by the model (Fig. 5). The use of LogK_OW_ as the input parameter of the Fish Plasma Model tended to overestimate the plasma BCF of the two compounds; whereas the use of LogD7.4 tended to underestimate it. The predictions generated in this work were based on the use of LogK_OW_; hence, it is plausible that our analysis overestimated the plasma concentrations of NSAIDs in fish. Nonetheless, this overestimation is in agreement with the precautionary principle that was applied throughout the workflow. The predictive model described here does not currently consider drug metabolism in fish, mainly due to the existing knowledge gaps in this field. Some studies have demonstrated that NSAIDs reactive metabolites may play a role in the manifestation of organ toxicity in mammalian models (Oda et al., 2017). However, the ecotoxicological relevance of those findings is currently unknown.

**Fig. 6:**
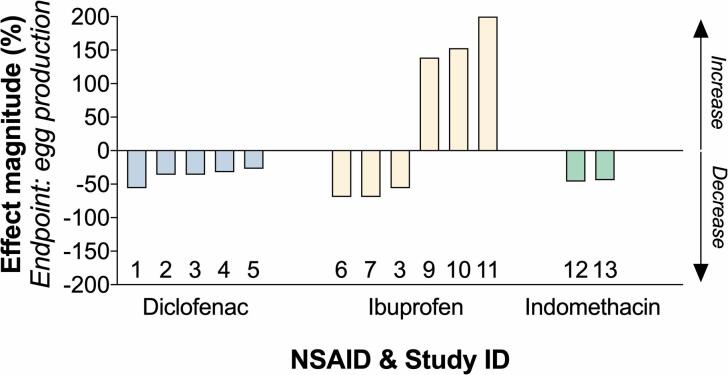
**Effect of NSAIDs on fish reproduction**. The figure displays effect magnitude and direction for the endpoint “egg production”. The data were retrieved from 6 studies published in the literature (1,2,4,5: Yokota et al., 2017; 3: Yokota et al., 2016; 6,8: Ji et al., 2013; 7,9,10: Han et al., 2010; 11: Flippin, Huggett and Foran, 2007; 12, 13: Lister and Van Der Kraak, 2008).

The interpretation of mode-of-action driven effects can be strengthened by the analysis of effect direction and magnitude. To assess this aspect, we retrospectively analysed those parameters for one of the endpoints with the highest regulatory importance, egg production (Fig. 6). Out of 13 experimental cases (retrieved from Flippin et al., 2007, Han et al., 2010, Ji et al., 2013, Lister and Van Der Kraak, 2008, Yokota et al., 2017; Yokota et al., 2015), NSAIDs (i.e. diclofenac, ibuprofen, indomethacin) induced a decrease in egg production in 10 cases, and an increase in 3 cases. Notably, the observed discrepancy was related to ibuprofen, with 3 cases of decrease and 3 cases of increase. The effect magnitude was 60% or lower in the cases of decreased egg production, and up to 200% in the cases of increased egg production. It is currently unknown if the observed discrepancy across the literature has a genuine biological explanation, or if it may be due to methodological artefacts.

## 4 Discussion

Implementing pharmacological and mechanistic considerations into the environmental risk assessment of pharmaceuticals can facilitate the interpretation of the risk and enable the application of modern predictive toxicology approaches. In the last decade, a number of experts have called for such implementation (e.g. Ågerstrand et al., 2015; Caldwell et al., 2014, Gunnarsson et al., 2019; Rand-Weaver et al., 2013; Winter et al., 2010). Several studies have experimentally demonstrated the positive impact of this approach (e.g. Margiotta-Casaluci et al., 2014, Margiotta-Casaluci et al., 2016, Valenti et al., 2012), and dedicated comparative pharmacology tools have been developed to facilitate the process (e.g. ECOdrug, Verbruggen et al., 2018; SeqPASS, LaLone et al., 2016). Despite these efforts, the current ERA process remains mechanistically agnostic and solidly centred on traditional fate/exposure predictions, with toxicity levels of individual compounds experimentally determined using simple tests focused on apical endpoints (Lee and Choi, 2019). This limitation acquires even more significance when mixtures of drugs are considered. In this case, the lack of mechanistic rationale behind the ERA of the individual components prevent the application of predictive approaches for the assessment of potential mixture effects. Moreover, the virtually endless number of exposure scenarios that may occur globally implies that the experimental determination of the risk is impossible to achieve, hence predictive approaches are vital to reach the desired future protection goals. The present work - focused on NSAIDs - paves the way for the development of an innovative pharmacology-informed ERA of drug mixtures by proposing a predictive framework that integrates both drug pharmacokinetic and pharmacodynamic features.

In Europe, the regulatory concern about NSAIDs has until now been focused on diclofenac and its effects on fish (European Commission, 2015, European Commission, 2018, Loos et al., 2018). A few academic studies have started to explore the potential effects of mixtures of NSAIDs, using a limited set of compounds - such as diclofenac, ibuprofen, naproxen, and aspirin - mostly using invertebrates (Cleuvers, 2004, Parolini and Binelli, 2012) or, when using fish, in combination with other chemicals (Parrott and Bennie, 2009; Stancova et al., 2014; Schmitz et al., 2018). Our analyses suggest that the problem of NSAIDs mixtures may be more significant than initially thought, as 19 out of 25 NSAIDs considered in the present study were detected in the aquatic environment worldwide. What is currently unknown is the level of co-occurrence of all these compounds in the same water body, as only a few of them are targeted in water monitoring programmes (Comber et al., 2018). Considering the impossibility of determining an average exposure scenario, in the present work we considered the mechanistic hazard of all 25 NSAIDs. The risk assessment, however, was performed using measured NSAIDs concentrations in the UK as the reference exposure scenario. This choice was justified by several factors. Firstly, the UK is characterised by a high market penetration of NSAIDs (McGettigan and Henry, 2013); secondly, the UK has one of the lowest average WWTP effluent dilution factors in the world (Keller et al., 2014); and thirdly, the UK carries out intensive environmental monitoring programmes that target pharmaceuticals (Comber et al., 2018). Nonetheless, the model presented here can be adapted to interpret the risk of any exposure scenario, once the concentrations of each component of the mixture are provided.

From a mechanistic standpoint, in a clinical context, the primary target of pharmaceuticals is generally involved in the disease pathophysiology, thus its modulation is aimed at achieving the desired therapeutic effect. Sometimes the interaction with the primary target is also the cause of adverse drug reactions. This is the case with NSAID-mediated COX-inhibition, which is considered the driving mechanism underlying many important side effects associated with NSAIDs treatment in patients (Grosser et al., 2017, Ricciotti and FitzGerald, 2011). However, in many other cases adverse drug reactions are driven by the unintended interaction of pharmaceuticals with secondary targets (Lounkine et al., 2012). From an ERA perspective, the exposure to pharmaceuticals is always unintended, thus the distinction between primary and secondary targets does not apply, and all drug-target interactions should be considered relevant for the mechanistic hazard assessment. This consideration led us to generate a bioactivity hazard network that captures the mechanistic promiscuity for a mixture of 25 NSAIDs, which was indeed significant in demonstrating that NSAIDs can act on many different targets beyond COXs (Fig. 2).

As expected, the complexity of the mechanistic network was drastically reduced when realistic internal exposure scenarios were considered (Fig. 3). Whereas some of the risk-based drug-target interactions were highly predictable (i.e. effects on COXs and interleukins) others were, to some extent, surprising. Specifically, the perturbation of estrogen and progesterone receptors (ESR1 and PGR) at concentrations of NSAIDs, respectively, hundreds and thousands of times lower than those predicted to occur in the plasma of wild fish in the UK. Previous research carried out on mammalian models has demonstrated that NSAIDs-mediated reduction of prostaglandin levels can lead to the down-regulation of the aromatase pathway and, in turn, decreased estrogen biosynthesis (Zhao et al., 1996). Diclofenac displayed anti-estrogenic activity at receptor level *in vitro* (Klopčič et al., 2018), whereas a study conducted on post-menopausal women also demonstrated that NSAID users had significantly lower serum estradiol concentrations than non-users (Hudson et al., 2008). On the other hand, the evidence of a direct link between NSAIDs and the progesterone receptor are scarcer. NSAIDs administration and NSAID-mediated prostaglandin decrease has been associated with the inhibition of ovulation in both pre-clinical mammalian species and humans (Gaytán et al., 2006; Stone et al., 2002), although the direct involvement of the progesterone receptor remains unclear. These considerations are relevant to the ERA of NSAIDs, as these compounds can also inhibit reproductive activity in female fish (Lister and Van Der Kraak, 2008, Yokota et al., 2016).

The AC50 value associated with the naproxen-mediated modulation of the PGR and the diclofenac-mediated modulation of the ESR1 were much lower than those associated with the inhibition of the drugs primary targets (COX-1 and COX-2). To facilitate the interpretation of their *in vivo* relevance, we compared those values with those associated with other potent pharmaceuticals that have the PGR and ESR as the primary targets. Runnalls et al. (2015) tested the effects of the synthetic progestin levonorgestrel (PGR agonist) and the synthetic estrogen ethinylestradiol (ESR agonist) on fish reproduction under chronic exposure conditions. Using these two compounds as the benchmark, it is possible to estimate the difference between the lowest ToxCast AC50 for the molecular initiating event and the drug plasma concentration that caused the statistically significant inhibition of egg production (effect size 30–40%). The latter was 3-fold higher for the levonorgestrel-PGR combination, and 10-fold higher for the ethinylestradiol-ESR combination. Based on these pharmacodynamic considerations, the reported ToxCast data for the interactions between naproxen-PGR and diclofenac-ESR would suggest that these compounds could cause PGR- and ESR-mediated reproductive effects at plasma concentrations of 5 ng/mL and 1,500 ng/mL, respectively. No reproductive toxicity studies have been carried for naproxen so far, but a few studies have been carried out with diclofenac. For example, Yokota et al. (2017) reported a water LOEC for reproductive effects of 37 µg/L diclofenac, corresponding to a predicted plasma concentration of 4500 ng/mL (prediction based on LogKow), which is only 3-fold higher than the above-mentioned prediction.

Collectively, this set of evidence indicate that pharmacodynamics-driven predictions may provide a valuable strategy to interpret the risk of mechanistic profiling data, although the inter-assay variability may represent a major confounding factor. For example, the ToxCast database contains 18 different assays which are able to the detect the perturbation of the PGR. Naproxen was only tested in 1 of those assays, in which it displayed positive activity. Similarly, 31 assays are available in the ToxCast database to detect the perturbation of the ESR1. Diclofenac was tested in 17 of those assays, displaying activity in 3, and inactivity in 14. In addition to the inter-assay variability issue, some authors have also raised concerns about the reliability of the nuclear receptor assays used in the ToxCast programme, and in turn, the reliability of their associated AC50 values. For example, Janesick et al. (2016) identified a high percentage of false positives among chemicals classified as PPARγ agonists in ToxCast. These considerations, together with the high inter-study variability observed for COXs IC50s (Supplementary Figure 2), reinforce the hypothesis that data generated from large-scale mechanistic profiling programmes are extremely valuable for generating testable hypotheses; whereas their direct application to drive the risk assessment process requires caution due to the high inter-assay variability of AC50s and IC50s.

Interpreting the *in vivo* relevance of the aforementioned *in vitro* mechanistic profiling data remains a major challenge in the field of toxicology. Corsi et al. (2019) tried to overcome this challenge by linking the ToxCast-informed bioactivity profile of a mixture of chemicals detected in the US Great Lakes with existing Adverse Outcome Pathways (AOPs). Furthermore, the ToxCast website itself links bioactivity data to existing AOPs whenever possible. In the present work, we observed that only a limited number of targets in our network was associated with AOPs in the AOPWiki. Although this approach may be a valuable strategy in the future, we concluded that the development stage of the AOPWiki is currently too preliminary to generate reliable *in vivo* predictions when applied to complex networks, such as the one generated for the 25 NSAIDs considered here. To overcome this challenge, we applied a different strategy by carrying out a zebrafish-specific target-to-phenotype association analysis for all those targets modulated at environmentally relevant concentrations of NSAIDs. This approach generated highly granular phenotypic predictions that could be used, for example, to guide the development of tailored *in vivo* experimental strategies.

Despite the successful application of the network pharmacology approach described here, there are some caveats that should be taken into consideration. Firstly, the NSAIDs bioactivity networks generated in this study are based on mammalian (largely human) data. Fish and human drug targets may display a different sensitivity to the same pharmaceutical compound. From a precautionary principle perspective, this factor may represent an issue only if the AC50s (or IC50s) for fish targets are significantly lower than the human ones; however, to our knowledge there is no evidence to support this hypothesis. A second limitation is that the target-to-phenotype association analysis is focused on zebrafish larvae and generates only qualitative predictions of the potential drug-induced phenotypes. These qualitative predictions cannot be used to infer effect magnitude, limiting the ability to directly inform the risk assessment process.

To overcome the latter limitation and provide a quantitative predictive model of NSAIDs-mediated effects in fish, we adopted a complementary predictive strategy centred on the primary targets of NSAIDs, rather than on their entire bioactivity network. In humans, NSAIDs exert their therapeutic action by inhibiting the enzymes COX-1 and/or COX-2, which are involved in the biotransformation of arachidonic acid into prostanoids. The biology of COXs and prostanoids has been extensively reviewed by many authors (e.g. Grosser et al., 2017, Ricciotti and FitzGerald, 2011), and it will not be discussed here. However, a basic comparative description of COXs functions in humans and fish is essential to appreciate the implications for the ERA of NSAIDs. COX‐1 is constitutively expressed in most tissues and is involved in basal production of prostanoids. The latter play important physiological functions, including gastric epithelial cytoprotection (Grosser et al., 2017). The perturbation of these physiological functions by non-selective NSAIDs may increase the risk of developing serious adverse effects, including gastrointestinal complications which are considered the most common NSAIDs-related adverse effects (Coxib and traditional NSAID Trialists' (CNT) Collaboration, 2013). On the other hand, COX‐2 is generally not expressed under basal conditions, but it is rapidly upregulated in response to inflammation, and its products (e.g. prostaglandin E2) potentiate the acute inflammatory response (Grosser et al., 2017). This mechanistic observation justified the development of COX-2 selective inhibitors (Fitzgerald and Patrono, 2001). Acting only (or mainly) on the inducible COX-2, this sub-class of NSAIDs is indeed associated with a lower risk of gastrointestinal toxicity in the majority of studies (Conaghan, 2012, García Rodríguez and Barreales Tolosa, 2007). However, after clinical approval, it rapidly emerged that COX-2 selective inhibitors were also associated with higher incidence of cardiovascular adverse events (Mukherjee et al., 2001). This unexpected scenario led to the withdrawal of rofecoxib and valdecoxib from the market in 2004 and 2005, respectively (Cotter and Wooltorton, 2005, Dieppe et al., 2004). However, other COX-2 selective inhibitors (e.g. celecoxib) continue to be used in the clinic. Follow-up research demonstrated that COX-2 is not only upregulated during inflammation but is also involved in the production of prostanoids with homeostatic functions under basal conditions. For example, gastrointestinal mucosa, vasculature, and brain tissue have all been show to express COX-2 in absence of inflammation (Grosser et al., 2017; Wallace and Devchand, 2005). COX-2-derived prostaglandin I2 and E2 are involved in the regulation of renal perfusion and blood pressure (Qi et al., 2002), and prostaglandin I2 is also involved in the antithrombotic mechanisms of the vessel wall (Grosser et al., 2006). These functions mechanistically explain the increased risk of cardiovascular adverse effects (Grosser et al., 2006). The review of the safety profile of NSAIDs has become the object of regulatory attention and frequent updates (for a review see Fanelli et al., 2017). For example, the US Food and Drug Administration requested a boxed warning concerning the cardiovascular risk of NSAIDs in 2005; this warning was strengthened in 2015 to highlight that all non-aspirin NSAIDs (both COX-2 selective and non-selective) can increase the risk of heart attack and stroke (US Food and Drug Administration, 2018).

The lesson learnt from the human safety assessment of NSAIDs suggests that any attempt to define clear-cut safe exposure levels of these compounds for fish, with the currently available relatively small body of evidence, may be over-ambitious. COX-1 and COX-2 are also expressed in the zebrafish, and COX inhibitors suppress the formation of prostaglandins *in vivo* (Grosser et al., 2002, Patel et al., 2016). However, the interpretation of the phenotypic relevance of COX inhibition in fish is complicated by the whole-genome duplication that occurred in the teleost lineage after its divergence from the tetrapod lineage (Taylor et al., 2003). Ishikawa et al. (2007) demonstrated that the genome of zebrafish contains two functional inducible isoforms of COX-2 genes, and that other fish species also contain alternate duplication and retention of COX-1 and COX-2. It is currently unknown if these duplication events also influence the species-specific pharmacological profile of NSAIDs. On the other hand, it is known that prostaglandins are involved in the regulation of regulatory-relevant phenotypes in teleost fish species, including development (Cha et al., 2006, Grosser et al., 2002) and reproduction (Stacey and Goetz, 1982, Takahashi et al., 2018), but also immunity (Gómez-Abellán and Sepulcre, 2016), kidney function, and gill function (Choe et al., 2006). Overall, this set of comparative pharmacological considerations justified the use of COX-1 inhibition as the key mechanistic parameter to interpret NSAIDs-mediated effects in our target-centred model.

From a mixture perspective, all NSAIDs act on COX-1 and COX-2, hence the most obvious approach was to consider the cumulative inhibition of the primary targets, especially COX-1, as the key event driving the toxicological risk. To do so, we expressed all NSAIDs in units of diclofenac-equivalents, using the diclofenac COX-1 IC50 as the reference value for the equivalence calculation. This approach is conceptually similar to the calculation of estrogenic equivalents to express mixtures of estrogenic chemicals (i.e. using the potency of 17-beta estradiol as the reference value; Safe, 1998). Brian et al. (2005) were the first to demonstrate that the estrogenic equivalence model can predict the response of fish to estrogenic chemicals. Our work advances this concept one-step forward by explicitly considering the drug concentrations in the fish plasma, rather than in the surrounding water. This shift from external to internal concentrations is essential to enhance the predictive power of the model, as previous studies have demonstrated that pharmaceuticals with comparable *in vitro* potency can lead to very different *in vivo* risk, based on their specific uptake and PK profile (Margiotta-Casaluci et al., 2016).

Integrating NSAIDs pharmacokinetic and pharmacodynamic considerations with the concept of pharmacological equivalence, we generated a powerful visual tool that summarises all the existing *in vivo* data concerning the chronic toxicity of NSAIDs in fish, as one single graph (Fig. 4). This analysis revealed that 30% of effect data points retrieved from the scientific literature were predicted to occur at concentrations lower than the worst-case exposure scenario in the UK (highest average measured NSAIDs concentrations in WWTP effluents), whereas this percentage dropped to 9% when a more realistic exposure scenario is considered (i.e. measured NSAIDs concentrations in surface waters). The latter sub-set of data was originated from 4 out of 26 *in vivo* studies considered in the present work (Ji et al., 2013, Mathias et al., 2018, Morthorst et al., 2018, Stancova et al., 2015). It is important to note that the proposed framework is a dynamic model that can be updated as and when additional biological data becomes available. Another key feature of the model is the potential to adapt the environmental exposure threshold to other exposure scenarios of interest, once the concentrations of the individual components of the NSAIDs mixture are known. This flexible approach can facilitate the region-specific interpretation of the toxicological risk posed to fish by NSAIDs locally, and effectively support regulatory decision-making.

The robustness of ERA is directly affected by the quality of the underlying data. In recent years, a growing number of authors have expressed concern about the degree of quality and reproducibility of ecotoxicology studies (Harris and Sumpter, 2015, Martin et al., 2019, Mebane et al., 2019). NSAIDs – specifically diclofenac – have been the object of intense debate due to discrepancies in toxicological and histopathological findings, observed between academic studies (Hoeger et al., 2005, Mehinto et al., 2010, Schwaiger et al., 2004, Triebskorn et al., 2004) and industry studies (Memmert et al., 2013). The controversy surrounding these discrepancies was fuelled by the fact that the outcomes of the four academic studies were used to justify the decision concerning diclofenac in Europe (i.e. its inclusion in the Watch List of emerging pollutants in 2015). A pathology working group was successively set up to independently review the histological sections from three of the studies that investigated the effects of diclofenac in trout (Hoeger et al., 2005, Mehinto et al., 2010, Memmert et al., 2013, Wolf et al., 2014). The pathology working group revealed that while some of the observed inter-study discrepancies were potentially due to the different experimental designs used in each study; the majority of inter-study variation was driven by issues of diagnostic interpretation (Wolf et al., 2014). Some discrepancies have also been observed for ibuprofen. In this case, its impact on fish reproduction has been highlighted as of concern, with a lowest observed effect concentrations (LOEC) (i.e. for zebrafish) as low as 1 µg/L (Ji et al. 2013). On the other hand, Morthorst et al. (2013) observed no effects on zebrafish egg production up to 506 µg/L (Morthorst et al., 2013), whereas a recent zebrafish short-term reproduction test set the LOEC, for the same endpoint, at 266 µg/L (Constantine et al., 2020). Overall, the discrepancies discussed above represent a challenge for regulatory decision-making. Our model does not contain a quality assessment of each study included in the analysis, however, this assessment could be carried out retrospectively by the end-user. This decision was justified by several reasons including the difficulty to set a univocal definition of ‘quality’ applicable to any context (e.g. academic vs industry, exploratory vs regulatory toxicology, etc.), and the risk of introducing undesired bias into the dataset. To demonstrate the positive value of retrospective analysis of specific data points in the model, we focused on one of the endpoints with the highest regulatory importance - egg production. We evaluated two important quantitative parameters: effect magnitude and effect direction. This analysis revealed a certain degree of inconsistency in the effects induced by ibuprofen, which sometimes caused a decrease in egg production and other times an increase. It is currently unknown if the observed discrepancy has a genuine biological explanation, or if it may be due to methodological artefacts. In any case, it suggests that this type of evaluation should be taken into consideration during the risk assessment process.

It is important to consider that the human safety assessment of NSAIDs so far has been based on the results of hundreds of studies. For example, the meta-analysis published by the Coxib and traditional NSAID Trialists' (CNT) in 2013 identified 754 randomised trials involving more than 350,000 patients. Despite these numbers, the interpretation of the risk remains complex and the discussion remains open (Coxib and traditional NSAID Trialists' (CNT) Collaboration, 2013). As a term of comparison, our COX-1-centred model portrays almost all existing data (to our knowledge) concerning the medium-to-long term effects of NSAIDs on fish. The model is based on 26 independent studies involving approximately 6,000 fish of several species at various life stages (mostly at early life stage). This number of animals already used to investigate the risk posed by NSAIDs in the aquatic environment is not negligible and raises the question whether additional *in vivo* ecotoxicity testing is needed. Our approach maximises the value of each *in vivo* study by integrating all data within a coherent predictive toxicology framework. For example, a very recent zebrafish short-term reproduction test involving 280 adult animals was carried out by Constantine et al. (2020). This study was not included in our dataset and it was used to test the degree of concordance with the model displayed in Fig. 4. Constantine et al. (2020) showed that 55 and 266 μg/L of ibuprofen caused 38% and 96% decrease of cumulative egg production, respectively (note: the effects at 55 μg/L were not statistically significant). Those exposure concentrations correspond to a plasma diclofenac-equivalents concentration of 53 and 258 ng/mL, which fall within the 30th percentile of the range of internal effect concentrations identified in our analysis (Fig. 4). This agreement highlights the high predictive value of our model and its potential to support weight-of-evidence driven regulatory decision making.

## 5 Conclusions

In the present study we provide a pharmacology-informed workflow able to guide the incorporation of pharmacokinetics and pharmacodynamic considerations into the environmental risk assessment of NSAIDs and aid the implementation of predictive toxicology strategies, without the immediate need of performing additional animal testing. Our analyses highlighted that 19 out of 25 NSAIDs have been detected in the aquatic environment globally, and demonstrated that the risk posed to fish by NSAIDs mixtures may not be negligible in situations of high population density (corresponding to high levels of drug consumption) and low dilution of WWTP effluents. Using the concept of pharmacological equivalence, we generated a multi-scale model able to guide the interpretation of the toxicological relevance of any given set of environmental concentrations of NSAIDs. We anticipate that this model could facilitate the interpretation of complex data and guide the regulatory decision-making process to better address the issue of both single NSAID and NSAIDs mixtures in the environment. On the other hand, the mechanistic, pharmacological, and biological complexity brought to light by the present work suggests that the clinical substitution of one NSAID with another - on the basis of the potential environmental risk - is far from simple and could have negative clinical implications, for example, by limiting the range of therapeutic options available to patients for the treatment of pain and inflammation.

## Funding

This work was funded by a Biotechnology and Biological Sciences Research Council (BBSRC) Research Grant (BB/P505018/1), co-funded by the AstraZeneca Global Safety, Health and Environment research programme, to L.M-C. supporting P.M.

## CRediT authorship contribution statement

**Philip Marmon:** Data curation, Formal analysis, Investigation, Methodology, Visualization, Writing - original draft, Conceptualization, Validation, Writing - review & editing. **Stewart F. Owen:** Funding acquisition, Supervision, Conceptualization, Validation, Writing - review & editing. **Luigi Margiotta-Casaluci:** Project administration, Resources, Data curation, Formal analysis, Investigation, Methodology, Visualization, Writing - original draft, Funding acquisition, Supervision, Conceptualization, Validation, Writing - review & editing.

## Declaration of Competing Interest

The authors declare the following financial interests/personal relationships which may be considered as potential competing interests: This work was co-funded by the AstraZeneca Global Safety, Health and Environment research programme. S.F.O. is an employee of AstraZeneca, a biopharmaceutical company specialized in the discovery, development, manufacturing and marketing of prescription medicines. AstraZeneca provided support in the form of salaries for author S.F.O., and supporting grant to P.M., but did not have any additional role in the study design, data collection and analysis, decision to publish, or preparation of the manuscript.
