# Supplementary material for: Pharmacology-informed prediction of the risk posed to fish by mixtures of non-steroidal anti-inflammatory drugs (NSAIDs) in the environment
Source: Environ Int. 2021 Jan;146:106222. doi: 10.1016/j.envint.2020.106222 (PMC7786791; doi:10.1016/j.envint.2020.106222)
Supplement: Supplementary tables and figures [file mmc2.pdf]

Pharmacology-informed prediction of the risk posed to fish by mixtures of non-steroidal anti-inflammatory drugs (NSAIDs) in the environment

*Philip Marmon[1], Stewart F. Owen[2], Luigi Margiotta-Casaluci[1]**

[1]*Department of Life Sciences, College of Health, Medicine, and Life Sciences, Brunel University London, London, UB8 3PH, UK*

[2]*AstraZeneca, Global Environment, Alderley Park, Macclesfield, Cheshire, SK10 4TF, UK*

**Corresponding author:* Luigi.Margiotta-Casaluci@brunel.ac.uk

SUPPLEMENTARY TABLES AND FIGURES

Supplementary Table 1. Average measured environmental concentrations of NSAIDs in the UK and corresponding predicted plasma concentrations in wild fish. This specific exposure scenario was used to generate the toxicological predictions described in the present study.

Drug	Environmental concentration 1 (UK, µg/L)	Environmental matrix 1	Predicted fish plasma concentration 1 (UK, ng/mL)*, **	Environmental concentration 2 (UK, µg/L)	Environmental matrix 2	Predicted fish plasma concentration 2 (UK, ng/mL)*, **
Aspirin	0.0064	Surface waters	0.01	0.0235	WWTP (treated) effluent	0.02
Carprofen	Not detected	/	0	Not detected	/	0
Celecoxib	Not detected	/	0	Not detected	/	0
Diclofenac	0.04	Surface waters	4.71	0.42	WWTP (treated) effluent	50.71
Etodolac	Not detected	/	0	Not detected	/	0
Etoricoxib	Not detected	/	0	Not detected	/	0
Flufenamic acid	Not detected	/	0	Not detected	/	0
Flurbiprofen	Not detected	/	0	Not detected	/	0
Ibuprofen	0.03	Surface waters	1.76	0.94	WWTP (treated) effluent	64.36
Indomethacin	0.009	Surface waters	0.22	0.02	WWTP (treated) effluent	0.47
Ketoprofen	0.006	Surface waters	0.09	0.017	WWTP (treated) effluent	0.25
Ketorolac	Not detected	/	0	Not detected	/	0
Mefenamic acid	0.007	Surface waters	7.18	0.05	WWTP (treated) effluent	51.27
Meloxicam	Not detected	/	0	Not detected	/	0
Naproxen	0.047	Surface waters	0.95	1.23	WWTP (treated) effluent	25.07
Niflumic acid	Not detected	/	0	Not detected	/	0
Nimesulide	Not detected	/	0	Not detected	/	0
Oxaprozin	Not detected	/	0	Not detected	/	0
Piroxicam	Not detected	/	0	Not detected	/	0
Rofecoxib	Not detected	/	0	Not detected	/	0
Sulindac	Not detected	/	0	Not detected	/	0
Tenoxicam	Not detected	/	0	Not detected	/	0
Tolfenamic acid	Not detected	/	0	Not detected	/	0
Valdecoxib	Not detected	/	0	Not detected	/	0

*WWTP=waste-water treatment plant. * Data retrieved from the German Environmental Agency (UBA) database "Pharmaceuticals in the Environment" (Aus der Beek et al., 2016). ** Predicted using the fish plasma model, as described by Margiotta-Casaluci et al. (2014).*

Supplementary Figure 1

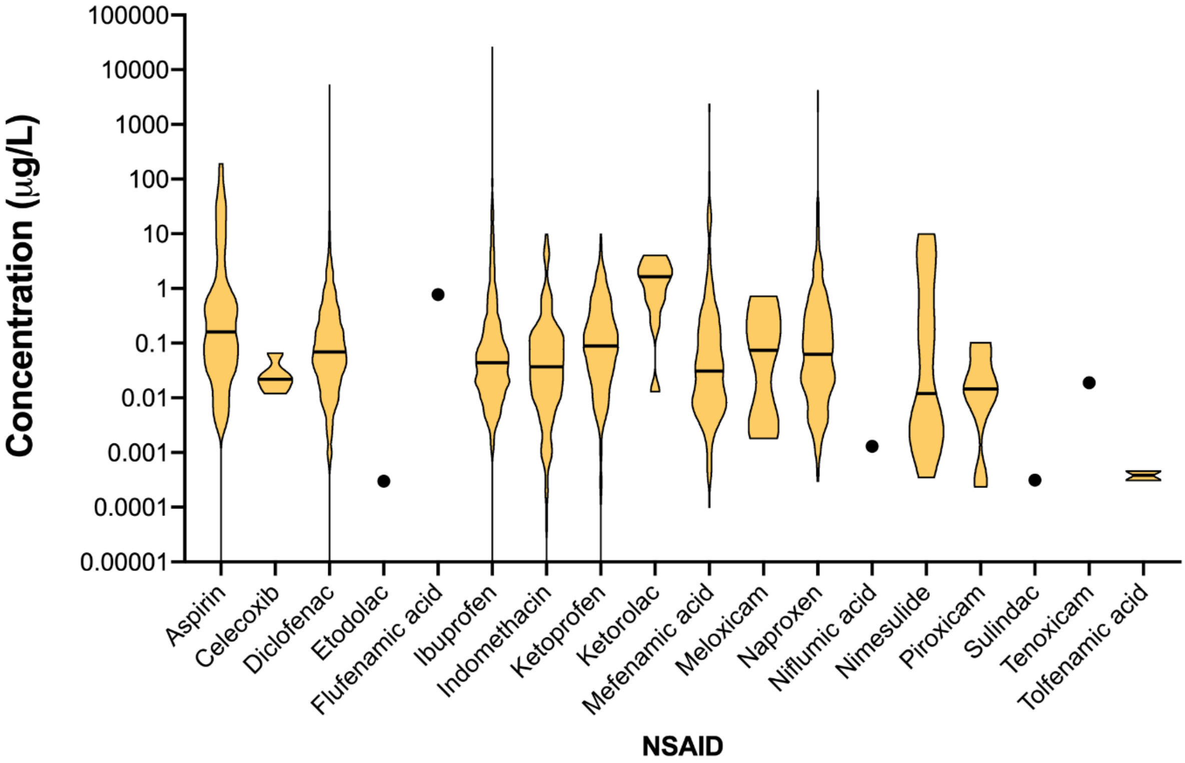

Supplementary Figure 1. Range of measured concentrations (µg/L) in surface waters and waste-water treatment plant effluents of 66 countries. The data was retrieved from the German Environmental Agency (UBA) database "Pharmaceuticals in the Environment" (der Beek et al., 2015). The line within each violin plot represents the median value. Only one concentration value was available for etodolac, flufenamic acid, niflumic acid, sulindac, tenoxicam. Each value is represented by a black dot.

Supplementary Figure 2

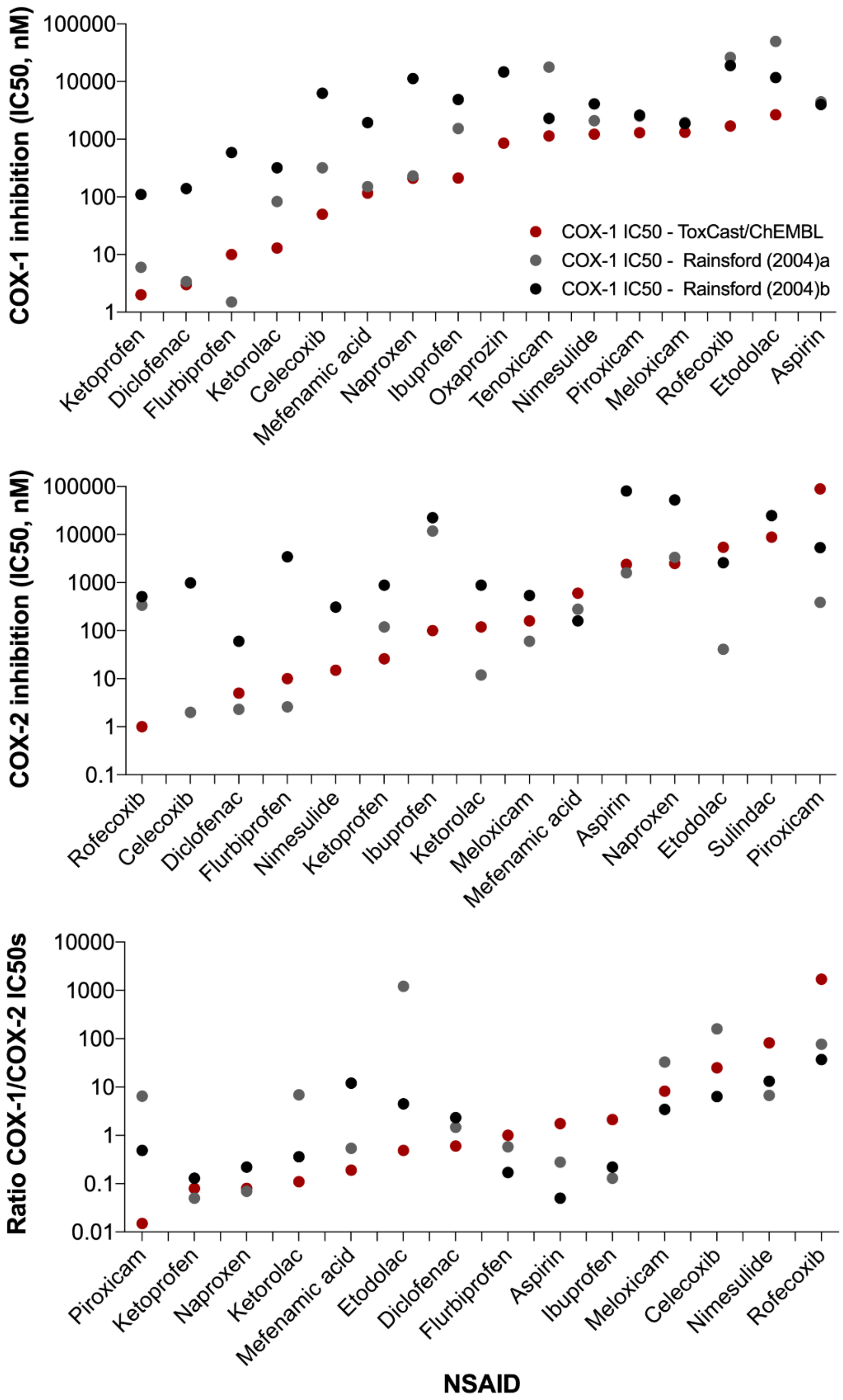

Supplementary Figure 2. Analysis of the variability of IC50 values retrieved from multiple sources (ToxCast/ChEMBL and Rainsford 2004). Note that the AC50 or IC50 values used in the present analysis were selected to be the lowest available in the database, in accordance with the precautionary principle. The three panels display the variability in A) COX-1 inhibition IC50s; B) COX-2 inhibition IC50s; C) COX-1/COX-2 inhibition ratio. The IC50 values labelled as "Rainsford (2004)a" were generated using human recombinant enzymes, whereas those labelled as "Rainsford (2004)b" were generated using whole human blood.

Supplementary Figure 3

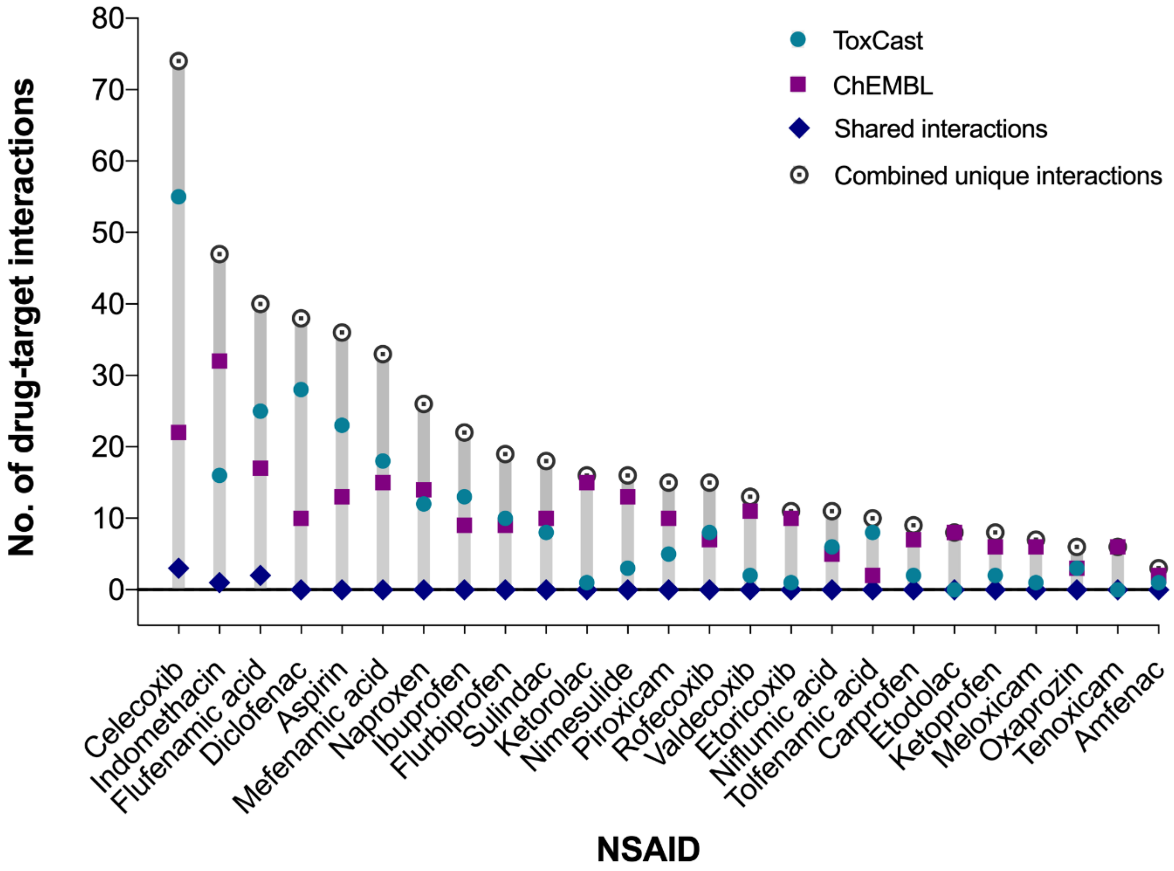

Supplementary Figure 3. Effect of ToxCast and ChEMBL data integration on the biological space covered by the bioactivity network. Note the minimal overlap between ToxCast and ChEMBL datasets (i.e. blue diamonds close, or equal to, zero).

Supplementary Figure 4

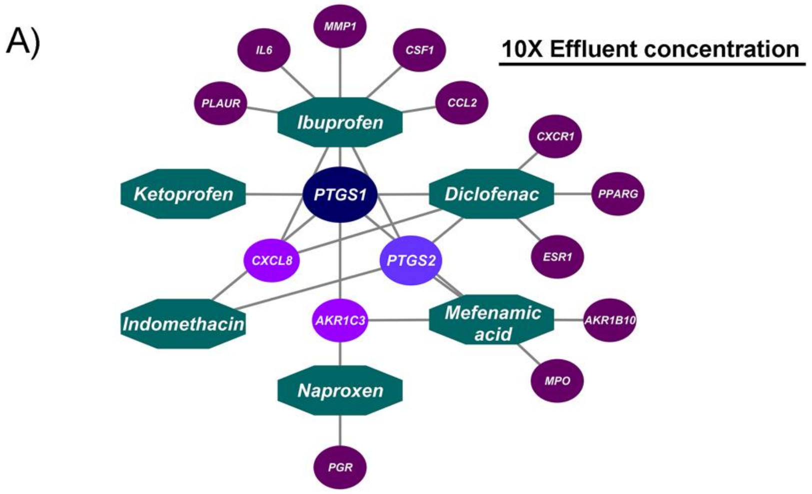

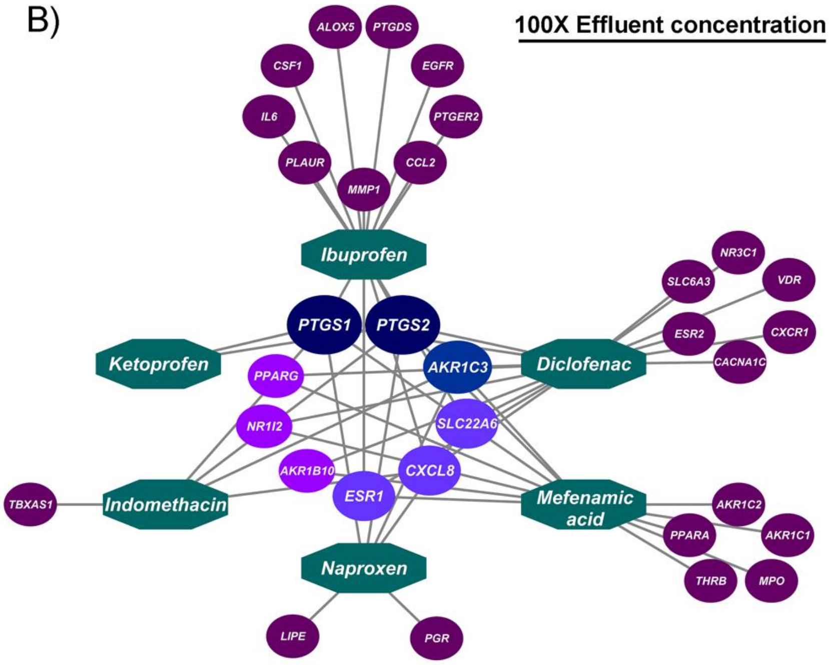

Supplementary Figure 4. Drug-target interaction networks for a mixture of 25 NSAIDs. The interactions were predicted to occur at (A) 10-fold above UK highest average measured concentration in wastewater treatment plant effluents, and (B) 100-fold above UK highest average measured concentration in wastewater treatment plant effluents. The green octagons indicate the single drugs. Drug targets are represented by color-coded nodes. Each colour indicates the different number of drugs that act on the associated target. Abbreviations: AKR1B10: Aldo-Keto Reductase Family 1 Member B10; AKR1C1: Aldo-Keto Reductase Family 1 Member C1; AKR1C2: Aldo-Keto Reductase Family 1 Member C2; AKR1C3: Aldo-Keto Reductase Family 1 Member C3; ALOX5: Arachidonate 5-Lipoxygenase; CACNA1C: Calcium Voltage-Gated Channel Subunit Alpha1 C; CCL2: C-C Motif Chemokine Ligand 2; CSF1: Colony Stimulating Factor 1; CXCL8: C-X-C Motif Chemokine Ligand 8; CXCR1: C-X-C Motif Chemokine Receptor 1; EGFR: Epidermal Growth Factor Receptor; ESR1: Estrogen Receptor 1; ESR2: Estrogen Receptor 2; IL6: Interleukin 6; LIPE: Lipase E, Hormone Sensitive Type; MMP1: Matrix Metallopeptidase 1; MPO: Myeloperoxidase; NR1I2: Nuclear Receptor Subfamily 1 Group I Member 2; NR3C1: Nuclear Receptor Subfamily 3 Group C Member 1; PGR: Progesterone Receptor; PLAUR: Plasminogen Activator, Urokinase Receptor; PPARA: Peroxisome Proliferator Activated Receptor Alpha; PPARG: Peroxisome Proliferator Activated Receptor Gamma; PTGDS: Prostaglandin D2 Synthase; PTGER2: Prostaglandin E Receptor 2; PTGS1: Prostaglandin-Endoperoxide Synthase 1; PTGS2: Prostaglandin-Endoperoxide Synthase 2; SLC6A3: Solute Carrier Family 6 Member 3; SLC22A6: Solute Carrier Family 22 Member 6; TBXAS1: Thromboxane A Synthase 1; THRB: Thyroid Hormone Receptor Beta; VDR: Vitamin D Receptor.
